# Genome-Wide Association Study for Test-Day Milk Yield, Proteins, and Composition Traits of Crossbred Dairy Cattle in Ethiopia

**DOI:** 10.1155/2024/1472779

**Published:** 2024-10-21

**Authors:** B. Rekik, T. Mestawet, A. Girma, M. Seid, J. Besufekad, S. Meseret

**Affiliations:** ^1^Department of Animal Science, College of Agriculture and Natural Resource Sciences, Debre Berhan University, PO Box 445, Debre Berhan, Ethiopia; ^2^School of Animal and Range Sciences, College of Agriculture, Hawassa University, PO Box 5, Hawassa, Ethiopia; ^3^Bio and Emerging Technology Institute (BETin), Addis Ababa, Ethiopia; ^4^Livestock Development Institute (LDI), Addis Ababa, Ethiopia; ^5^Livestock Genetics, International Livestock Research Institute (ILRI), Addis Ababa, Ethiopia

**Keywords:** genetic marker, genomic regions, GWAS, milk composition, milk yield, SNP

## Abstract

Identifying genetic regions and candidate genes that influence milk production traits is critical for understanding genetic inheritance and improving both the quality and quantity of milk in dairy cattle. Crossbred dairy cattle significantly contribute to increasing milk production and ensuring food security in the middle- and high-altitude regions of Ethiopia. However, the genetic architecture underlying their milk yield and composition traits has not yet been thoroughly investigated. This study conducted a genome-wide association study (GWAS) on 308 crossbred dairy cows from central, northeastern, and southern Ethiopia to identify genetic markers associated with key milk production traits. Using high-density SNP chip data and the fixed and random model circulating probability unification (Farm CPU) method via the Memory-efficient, Visualization-enhanced, and Parallel-accelerated R package (rMVP) (Version 1.0.7.), we analyzed traits including test-day milk yield (TDMY), total protein (TP), casein (CN), whey (W), protein percentage (P), fat percentage (F), lactose percentage (L), total solids (TS), density (D), solids-not-fat (SNF), salt (S), and freezing point (FP). This study identified 16 significant SNPs associated with these traits, including rs41661899 on Chromosome 6, which was significantly associated with both TP and W, and rs42274954 on Chromosome 12, which was significantly associated with CN. Eight SNPs, such as rs43560693, rs109098713, rs111029661, rs134499665, rs133908307, rs133627532, rs42098411, and rs110066280, were found across multiple chromosomes (8, 10, 14, 15, 19, 21, 26, and 28, respectively) and were significantly associated with milk P. Additionally, SNPs rs110844447 and rs135995768 on Chromosomes 6 and 14 were significantly associated with D and FP, respectively. Three SNPs, including rs109564259, rs135552551, and rs41620904 on Chromosomes 6, 11, and 24, were significant associations with S. Candidate genes identified near and within these SNPs include TRAM1L1, DIAPH3, PEBP4, WDR89, BCAS3, RALGAPA1, HABP2, NRG3, HPSE, PCDH7, LINC02579, TRNAS-GGA, and OR5CN1P. These findings enhance our understanding of the genetic architecture of milk-related traits in Ethiopian dairy cattle and highlight the potential for marker-assisted selection to improve milk production and composition in breeding programs.

## 1. Introduction

Dairy cattle in Ethiopia play a crucial role as the primary source of milk, significantly contributing to food security and income generation. The country's diverse agroecological zones offer substantial opportunities to enhance milk yield and composition by exploiting the genetic potential of various cattle breeds [1, 2]. Milk is a vital source of nutrition for both rural and urban populations in Ethiopia, providing key nutrients such as protein, calcium, vitamins, and fats [3]. However, the increasing demand for milk and milk products, driven by population growth, urbanization, and evolving dietary preferences, has created an urgent need to improve both the quantity and quality of milk produced by Ethiopian dairy herds [4, 5].

The composition of milk significantly impacts its processing characteristics and the quality of the dairy products. Major milk proteins, including casein (CN) and whey (W), play a central role in determining the suitability of milk for dairy processing, particularly in the production of yogurt and cheese [6]. CNs, in particular, influence the texture and stability of these products [7, 8], while W proteins are valued for their functional properties and nutritional significance [9]. Beyond their technological value, milk proteins are crucial in human nutrition, providing essential amino acids and bioactive peptides that exhibit antimicrobial [10–15] and antiviral activities [16–18]. Proteins also act as enzymes or hormones and serve many physiological activities [19]. These properties are influenced by the inherent composition of milk, which is shaped by factors such as animal genetics, feeds and nutrition, seasonality, lactation stage, lactation number, and cow health [20–27].

Crossbreeding in Ethiopia dairy farming is a common practice aimed at improving dairy cattle performance by combining the desirable traits of different breeds to achieve higher milk yields and superior milk composition [28, 29]. This typically involves crossbreeding indigenous Zebu cattle with exotic dairy breeds like Holstein Friesian or Jersey [29]. The genetic basis of milk yield and composition traits is complex, involving multiple genes with small to moderate effects as well as significant environmental influences [30]. Direct selection based on phenotypic data alone often results in limited improvements. Therefore, a deeper understanding of the molecular mechanisms influencing these traits is essential for significant progress in dairy cattle [31].

Genome-wide association studies (GWASs) are a powerful tool for understanding the genetic basis of complex traits, such as milk yield and composition, in dairy cattle. GWAS allows for the detection of single nucleotide polymorphisms (SNPs) associated with desirable traits by scanning the genome, providing insights into the genetic architecture of these traits, and facilitating the development of genomic selection tool variations [32, 33]. SNPs are the most abundant form of genetic variation and serve as valuable markers for identifying genetic regions associated with milk production traits. By leveraging these associations, breeding programs can implement genomic selection strategies to produce healthier, more productive dairy cattle [34–37].

Despite the extensive global research on SNPs related to milk traits in different dairy cattle breeds, comprehensive GWAS on milk-related traits in Ethiopian crossbred dairy cattle are lacking. This study is aimed at addressing this gap by identifying valuable genetic markers that can be used in marker-assisted selection and breeding programs to improve milk quality and yield in Ethiopian crossbred dairy cattle.

## 2. Material and Methods

### 2.1. Animals and Phenotypes

This study was conducted on 420 crossbred dairy cows from urban and periurban dairy farms located in central, northeastern, and southern Ethiopia. These regions were selected due to their significant contributions to the national dairy industry and their varying agroecological conditions, which provide a representative sample of the country's dairy production environment. The cows were fed a diet consisting of pasture, hay, and a formulated concentrate mix containing wheat bran (Triticum vulgare), nug cake (Guizotia abyssinica),and salt (S). The amount of concentrate was adjusted based on each cow's weight, milk yield, and lactation status. Farm records provided additional data on calving history, lactation length, lactation number, estimated exotic breed composition, and management practices. Holstein Friesian and Jersey are the primary sources of exotic ancestry in Ethiopia, with the exotic blood level of crossbred dairy cows being largely influenced by Holstein Friesian and, to a lesser extent, Jersey breeds. These breeds have long been imported into the country, either as live animals or semen for artificial insemination (AI), to improve the local cattle breeds.

The data were categorized into four genetic groups based on the exotic blood level and its distribution: ≤ 50%, 50.5%–75%, 75.5%–87.5%, and ≥ 88%. Lactation stages were classified as early (≤ 100 days), mid (101–200 days), late (201–305 days), and extended lactation (> 305 days), based on days in milk during sampling day. Lactation numbers were divided into four categories: 1st, 2nd, 3rd, and ≥ 4th based on the number of times a cow has produced milk. Calving seasons were grouped into three categories: long rainy, short rainy, and dry based on weather patterns. Calving years were categorized into 2020, 2021, and 2022.

A total of 5040 phenotypic records were collected on milk production traits, including test-day milk yield (TDMY), total protein (TP), CN, W, protein percentage (P), fat percentage (F), lactose percentage (L), total solids (TSs), density (D), solids-not-fat (SNF), S, and freezing point (FP). Milk yield was measured during both morning and evening milking sessions using graduated buckets or cups from each cow on the sampling day. The recorded morning and evening milk yields were summed to obtain the test-day daily milk yield.

Milk composition was analyzed using a near-infrared ultrasonic milk analyzer (Milkotronic Ltd.), a widely accepted device in dairy research for its accuracy and efficiency in measuring multiple milk components including fat, protein, lactose, TSs, D, SNF, FP, and S. The TP concentration of milk was determined using the Bradford method with Coomassie blue reagent, a standard approach for quantifying protein in biological samples, with absorbance measurements at 595 nm using a microplate reader (Multikan GO) controlled by SkanIt RE 6.1.1 software. CN was analyzed by the isoelectric precipitation method, and W protein was calculated by subtracting TP from CN.

Phenotypic values were normalized using a two-step approach, which involved log transformation followed by standardization to a mean of zero and a standard deviation of one. This was done to correct for skewness in the data distribution, ensuring that the statistical analyses were performed on data meeting the assumptions of normality [38]. For additional information on the study animals, phenotypic data, and environmental conditions, refer to the previous study by Bekele et al. [39].

### 2.2. Genotype, Imputation, and Quality Control (QC)

Genotype data were obtained for 320 cows from the Livestock Development Institute (LDI), which were genotyped using two SNP panels: the Illumina Bovine SNP50K_v2 Bead Chip array (54,609 SNPs, used for genotyping 220 cows) and the Bead Chip Illumina SNP100K array (107,478 SNPs, used for genotyping 100 cows). The two panels were harmonized through genotype imputation using Beagle Version 5.3, as described in B. Browning, Zhou, and S. Browning [40], ensuring comparable SNP data across all cows. Rigorous QC procedures were implemented using PLINK Version 1.9 [41]. Animals with a missing genotype rate greater than 10% or a Mendelian SNP error rate greater than 2% were excluded. SNPs were discarded if they had a call rate below 90%, a minor allele frequency (MAF) below 1%, or if they significantly deviated from the Hardy–Weinberg equilibrium (*p* < 0.001). After QC, 308 cows and 85,127 SNPs remained for the GWAS.

### 2.3. GWAS Analysis

The GWAS was conducted using the Memory-efficient, Visualization-enhanced, and Parallel-accelerated R package (rMVP)Version 1.0.7, applying the fixed and random model circulating probability unification (Farm CPU) model [42]. The rMVP package includes intermediate analysis functions such as principal component (PC) analysis (PCA) and computation of the kinship matrix. The Farm CPU model was chosen for its ability to control population structure and relatedness, critical for avoiding both false positives and false negative associations in GWAS [43]. The Farm CPU model iteratively adjusts for fixed and random effects, improving the detection power of true genetic associations. Fixed effects in the model included two PCs derived from a PCA, genetic groups, calving years, calving seasons, lactation lengths, lactation numbers, and geographical farm locations. These factors were included to account for potential confounders that could bias the results [42, 44]. The kinship matrix was calculated using the VanRaden algorithm, which estimates genetic relatedness between individuals developed by VanRaden [45]. Incorporating this matrix helped control for familial relationships that could lead to spurious associations. The significance threshold for identifying SNPs was set using the Bonferroni correction (*p* ≤ 5.9 × 10^−7^ or 0.05/85127), a stringent threshold necessary to reduce Type I errors given the large number of SNPs tested.

The model can be represented as

 $$\begin{array}{c} y=XB+e \end{array}$$

where *X* is the design matrix containing the incidence matrices for all of the fixed effects and the SNPs, *B* is the vector of all fixed effects, including the effects of quantitative trait nucleotides (QTNs) and the SNPs, and *e* is the vector of residuals.

The covariance matrix is given by

 $$\begin{array}{c} \text{Cov}\left(y\right)=V=2K\sigma^{2}u+\sigma^{2}e \end{array}$$

where *K* is the kinship matrix, *σ*^2^*u* is the genetic variance, and *σ*^2^*e* is the residual variance.

### 2.4. Exploring Candidate Genes

SNP names were converted to rs IDs using the Animal Genome Project's SNP name-to-ID tool (https://www.animalgenome.org/bioinfo/tools/SNPnmids/). Candidate genes were located within 200 kb on either side of the significant SNPs for the 12 milk production traits in crossbred dairy cattle population were identified using the Ensembl database (https://www.Ensembl.org/index.ht ml/), the DAVID bioinformatics tool (https://david.nchttp://ifcrf.gov/), and the UCSC Genome Browser (https://genome.ucsc.edu/). In total, 14 candidate genes or their closest genomic neighbors were identified as associated with significant SNPs. However, the functions and biological pathways of these genes are not discussed in this paper. Future research will explore their complex roles.

## 3. Result

### 3.1. Phenotypic Data

Descriptive statistics for the 12 milk production traits, including TDMY, TP, CN, W, P, F, L, TSs, D, SNF, S, and FP, were generated for the genotyped lactating dairy cows and are presented in Table 1. To assess the normality of the data distribution, standard deviations, confidence intervals, and coefficients of variation were calculated. The Shapiro–Wilk test returned the highest *p* values for the normalized data, indicating an approximately normal distribution of the dependent variables (see Figure S1). As a result, the data are considered suitable for further genome-wide association analyses.

### 3.2. Population Stratification Assessment

**Figure 1:**
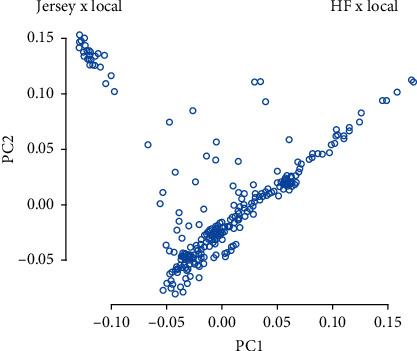
PCA plot demonstrating the population structure of 308 crossbred dairy cows. This PCA plot illustrates the genetic population structure of 308 crossbred dairy cows, highlighting the genetic difference between two major crossbred groups: Jersey × Local and Holstein Friesian (HF) × Local. Each point in the plot represents an individual cow, and the two groups are clearly separated, suggesting distinct genetic backgrounds. The first and second principal components (PC1 and PC2) explain the majority of the genetic variation, allowing for a clear distinction between the breeds.

PCA was performed using the 85,127 SNPs to explore the genetic structure of the 308 crossbred dairy cattle. The PCA scatter plot (Figure 1) illustrates the relationship between the first two PCs (PC1 and PC2), revealing a clear separation of cattle into two distinct clusters: “Jersey × Local” and “HF × Local.” This separation indicates significant genetic divergence between the two groups. The “Jersey × Local” cluster, located on the left side, is small and tightly grouped, suggesting few in number, and they had high genetic similarity, whereas the “HF × Local” cluster, on the right, is large and dispersed, shows large in number, and they had greater genetic diversity. These clusters reflect distinct genetic backgrounds, likely resulting from the crossbreeding of local cattle with Holstein Friesian and Jersey breeds.

### 3.3. SNP Distribution Across the Genome

**Figure 2:**
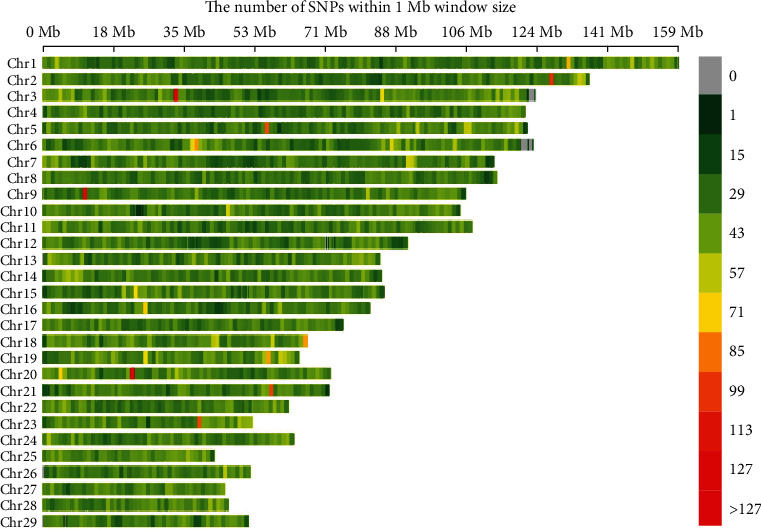
Genome-wide distribution of 85,127 SNP markers across 29 chromosomes. This figure displays the genome-wide distribution of 85,127 SNP markers across 29 autosomal chromosomes in 308 crossbred dairy cows. The *x*-axis represents the chromosomal positions of the SNPs, and the *y*-axis shows the SNP density at different positions along the chromosomes. SNPs are color-coded to reflect marker density.

The distribution of 85,127 SNP markers across the 29 chromosomes is presented in Figure 2 and Table S1. SNPs serve as genetic markers, and their distribution across the genome is essential for identifying genetic regions associated with milk production traits. They can be used to study genetic variation, evolution, and the function of different organisms. A 1 Mb gap is a segment of DNA containing one million base pairs that make up DNA. The legend color scale is used to indicate SNP markers in the genome with low densities represented by relatively green colors, high densities represented by red colors, and regions with no markers. SNP D is the number of SNP markers per 1 Mb interval. A SNP D plot can be used to compare SNP D across different chromosomes and regions within chromosomes. Some chromosomes had a large number of SNPs, and some had higher SNP D. Chromosome 1 exhibited the highest number, while Chromosome 27 had the fewest number of SNPs. Chromosome 19 showed the highest SNP D, whereas Chromosome 12 displayed the lowest. Notably, Chromosome 3 showed regions of high SNP D around the 35 Mb position and lower D around the 120 Mb position. These findings reveal regions of interest for further analysis, particularly where SNP D is either significantly higher or lower, as these may indicate genomic regions associated with milk production traits.

### 3.4. GWAS

**Figure 3:**
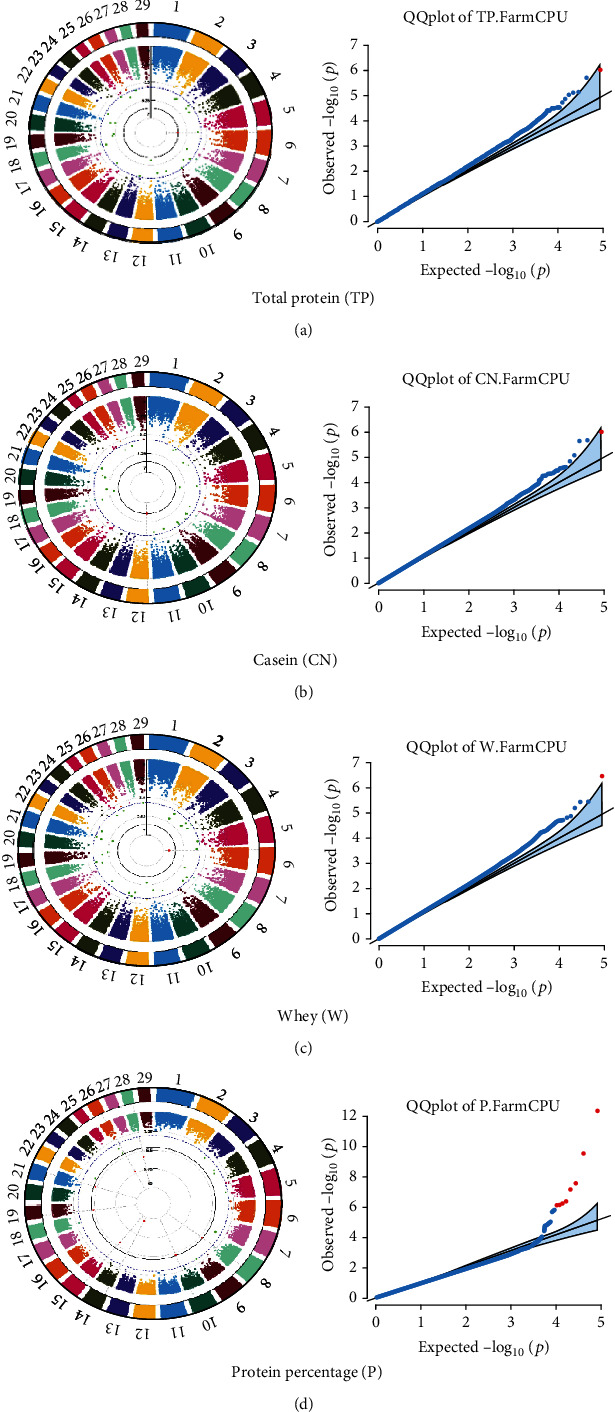
Circular Manhattan and quantile–quantile plots for four milk protein traits. This figure shows the circular Manhattan plots and quantile–quantile (QQ) plots for four key milk protein traits: (a) total protein, (b) casein, (c) whey, and (d) protein percentage. Red dots represent significant SNPs that pass the genome-wide significance threshold, with the black solid line indicating the cutoff for the statistical significance. The corresponding QQ plots (i–iv) show the observed versus expected *p* values for each trait, allowing for the identification of potential false positives in the Farm CPU model.

**Figure 4:**
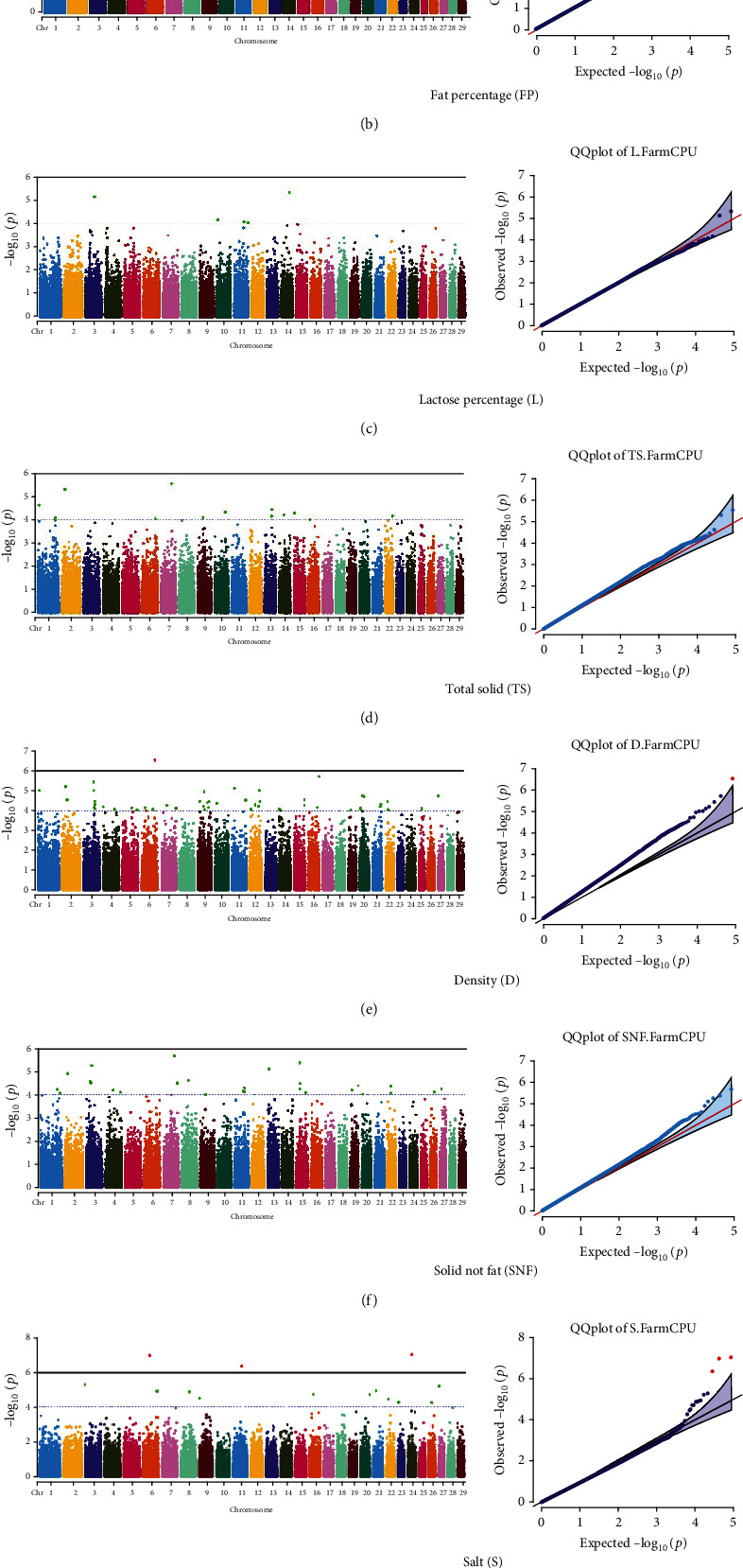
Rectangular Manhattan and quantile–quantile plots for milk production traits. This figure presents rectangular Manhattan plots and QQ plots for milk production traits, including (a) test-day milk yield, (b) fat percentage, (c) lactose percentage, (d) total solids, (e) density, (f) solids-not-fat, (g) salt, and (h) freezing point. The black line indicates the genome-wide significance threshold. Red dots represent significant SNPs, while green dots indicate suggestive SNPs. Roman numbers (I–VIII) correspond to the QQ plots, showing the observed versus expected *p* values for each trait, allowing for the identification of potential false positives in the Farm CPU model.

Quantile–quantile (QQ) plots (Figures 3 and 4) were generated for each of the 12 milk production traits to assess the distribution of *p* values and validate the GWAS results. Minimal deviations from the expected *p* values were observed for most traits, confirming the robustness of the GWAS model. Deviations from the null distribution were only apparent in the upper tails of the QQ plots, suggesting strong associations between SNPs and specific milk production traits.

The GWAS identified 16 significant SNPs associated with seven key milk production traits: TP, CN, W, P, D, S, and FP. These results are summarized in Table 2, with Manhattan plots (Figures 3 and 4) illustrating the SNP associations across the genome. The circular Manhattan plots in Figure 3 present a genome-wide overview of SNP associations for key milk protein traits (TP, CN, W, and P), while the rectangular Manhattan plots in Figure 4 display the associations for additional milk production traits, including TDMY, F, L, TS, D, SNF, S, and FP. The significant SNPs passing the genome-wide significance threshold (*p* ≤ 5.9 × 10^−7^) are indicated as red dots, highlighting loci of interest. Among these noteworthy findings are as follows: rs41661899 on Chromosome 6 was associated with both TP and W, with the nearest gene being TRAM1L1, as shown in Figures 3(a) and 3(c). rs42274954 on Chromosome 12 was associated with CN, located near the DIAPH3, as shown in Figure 3(b). A particularly significant association was found for P with eight SNPs identified, including rs43560693, rs109098713, rs111029661, rs134499665, rs133908307, rs133627532, rs42098411, and rs110066280, spanning multiple chromosomes (8, 10, 14, 15, 19, 21, 26, and 28, respectively), as shown in Figure 3(d). The candidate genes near these SNPs include PEBP4, WDR89, BCAS3, RALGAPA1, HABP2, and NRG3, as listed in Table 2. SNP rs110844447 on Chromosome 6 was significantly associated with milk D, with HPSE being the nearest gene, as shown in Figure 4(e) and Table 2. Three SNPs were associated with S content, including rs109564259 on Chromosome 6, with the nearby gene PCDH7; rs135552551 on Chromosome 11, with the nearest gene LINC02579; and rs41620904 on Chromosome 24, with the nearest gene TRNAS-GGA, as shown in Figure 4(g) and Table 2. Finally, SNP rs135995768 on Chromosome 14 with the nearest gene OR5CN1P, as shown in Figure 4(h) and Table 2.

Chromosome position (*x*-axis) versus −log10(*p*) (*y* − axis) scatter shows genome-wide associations of significant loci with milk production traits. The black line indicates the genome-wide significant threshold (*p* value ≤5.9 × 10^−7^), and the blue line indicates the genome-wide suggestive threshold (*p* value ≤4 × 10^−5^). The red dots represent SNPs that passed the genome-wide significant threshold (*p* value ≤5.9 × 10^−7^), and the green dots are SNPs that passed the suggestive threshold.

## 4. Discussion

Milk quantity and quality are essential traits in the dairy industry, with significant economic implications. Numerous genes and genomic regions have been studied for their associations with milk-related traits to enhance milk yield, composition, and other relevant characteristics [46]. The results of this GWAS provide novel insights into the genetic architecture of milk production traits in crossbred dairy cattle in Ethiopia, revealing significant associations between SNPs and key milk production traits. The application of the Farm CPU method enabled the identification of genetic markers with greater precision by controlling for population structure and relatedness [47]. This approach helped minimize false positives while maximizing the discovery of true genetic associations.

The PCA revealed a clear population structure within the cattle herds, grouping individuals based on their genetic characteristics (Figure 1). The experimental population clustered into two distinct groups, largely reflecting differences in exotic blood levels from Holstein Friesian and Jersey breeds. The larger group, primarily influenced by Holstein Friesian genetics, exhibited more genetic diversity, while the smaller cluster, with a higher concentration of Jersey influence, showed less diversity. This clustering of genetic groups underscores the importance of controlling for population structure in GWAS to avoid confounding effects [44, 48, 49].

### 4.1. Significant SNPs and Candidate Genes

This study identified 16 significant SNPs associated with seven milk production traits, including TP, CN, W protein, P, D, S, and FP. These associations point to important candidate genes that influence milk traits. SNP, rs41661899 on Chromosome 6, was significantly associated with both TP and W, with TRAM1L1 identified as the nearest candidate gene. Previous studies similarly reported the TRAM1L1 gene as significantly associated with milk-related traits and highlighted its role in milk protein synthesis [50–53]. The SNP, rs42274954 on Chromosome 12, associated with CN and located near the DIAPH3 gene, underscores the genetic influence on CN levels, which are crucial for cheese production and other dairy processing qualities. DIAPH3 has been previously reported to affect protein assembly or secretion in mammary tissues [54]. The identification of eight SNPs associated with P is particularly important for the dairy industry, as protein content determines the quality and yield of processed dairy products such as cheese and yogurt. The most significant SNP, rs43560693, located near the PEBP4 gene on Chromosome Number 8, was associated with P. This finding is consistent with Kim et al. [55], who reported PEBP4 as a candidate gene associated with milk and fat yield in Koran Holstein cattle. PEBP4 has also been noted in several studies for its association with milk-related and carcass traits [56–58]. The SNP, rs109098713, located near the WDR89 gene on Chromosome 10, was associated with P. Sutera [59] reported that WDR89 is associated with milk protein in Valle del Belice dairy sheep. The SNP, rs111029661 on Chromosome 14, was identified as a significant genetic marker for milk P, although no nearby genes were found within 200 kb. Interestingly, Chromosome 14 has been associated with several milk production traits [60]. The SNP, rs134499665 on Chromosome 15, exhibited a significant association with milk P, though no nearest candidate gene was identified within 200 kb. The SNP, rs133908307 on Chromosome 19, located near BCAS3, was significantly associated with milk P. BCAS3 has been reported as a candidate gene for milk technological traits in Assaf and Churra dairy breeds and has also been reported for milk production and conformation traits in Canadian Alpine and Saanen dairy goats [61]. The SNP, rs133627532 on Chromosome 6, near the RALGAPA1 gene, was associated with P in this study. A previous study similarly reported that the RALGAPA1 gene is associated with milk-related traits in Sahiwal cattle [62]. The SNP, rs42098411 on Chromosome 26, located on the HABP2 gene, was associated with P. Similarly, the previous study reported that HABP2 was associated with milk traits [63]. Furthermore, SNP, rs110066280 on Chromosome 28, located on the NRG3 gene, was associated with P in this study, consistent with earlier studies reporting the NRG3 genes associated with P and milk yield in dairy sheep and buffalo [64, 65].

This study identified three novel SNPs significantly associated with S content: rs109564259 on Chromosome 6 (nearest PCDH7), rs135552551 on Chromosome 11 (nearest LINC02579), and rs41620904 on Chromosome 24 (nearest TRNAS-GGA). S content in milk is an important factor influencing taste and dairy processing. These findings introduce new candidate genes that may regulate the mineral content of milk, opening new avenues for genetic selection aimed at improving milk quality. An et al. [66] previously reported PCDH7 associated with body size traits in Simmental beef cattle. S content in milk is an important factor influencing taste and dairy processing. These findings introduce new candidate genes that may regulate the mineral content of milk, opening new avenues for genetic selection aimed at improving milk quality. Moreover, SNP rs135995768 on Chromosome 14 was significantly associated with FP, with OR5CN1P identified as the nearest pseudogene, suggesting that even noncoding regions of the genome may have regulatory roles that affect milk quality. FP is critical for milk storage, and understanding its genetic basis could lead to improved storage and processing practices.

## 5. Conclusion

This GWAS on TDMY, protein, and composition traits in crossbred dairy cattle in Ethiopia marks a pivotal step forward in advancing the country's dairy sector. By identifying 16 significant SNPs associated with key milk production traits, we provide valuable genetic markers that can be utilized in marker-assisted selection and breeding programs to improve milk quality and yield. The identification of candidate genes, such as TRAM1L1, DIAPH3, and PEBP4, offers critical insights into the genetic architecture influencing these traits. These findings underscore the importance of targeted breeding strategies that integrate genomic data to enhance the potential dairy cattle. In particular, the significant associations with protein content, S, and FP provide actionable insights into improving both the quantity and quality of milk in Ethiopian crossbred cattle. This study establishes a foundation for further studies integrating genomic data into breeding programs and achieving significant genetic gains, contributing to both food security and the economic well-being of smallholder dairy farmers. Future research should aim to expand the sample size and validate the identified SNPs in different cattle populations. Incorporating environmental and management factors, as well as exploring gene-environment interactions, will be critical to refining genetic models and improving the accuracy of breeding predictions. By continuing to invest in genomic research and breeding technologies, Ethiopia has the opportunity to revolutionize its dairy industry and meet the increasing demand for high-quality milk and dairy products.

## Figures and Tables

**Table 1:** Descriptive statistics of the milk production traits in crossbred genotyped dairy cows (*n* = 308).

**Traits**	**Mean**	**Min**	**Max**	**SD**	**CV (%)**
Test-day milk yield (TDMY)	14.77	5.30	22.50	3.96	26.80
Total protein (TP)	35.51	15.64	59.21	8.80	24.80
Casein (CN)	29.28	15.09	45.20	6.27	21.40
Whey (W)	6.23	0.64	12.71	2.52	40.50
Protein percentage (P)	3.24	2.10	4.37	0.41	12.70
Fat percentage (F)	3.10	0.90	5.32	0.87	28.10
Lactose percentage (L)	4.50	3.03	5.92	0.56	12.40
Total solids (TSs)	10.86	9.16	12.56	0.65	6.00
Density (D)	29.19	17.76	41.82	4.91	16.80
Solids-not-fat (SNF)	7.77	4.26	10.98	1.30	16.80
Salt (S)	0.65	0.46	0.86	0.08	12.60
Salt (FP)	−0.50	−0.66	−0.34	0.07	

**Table 2:** The significant SNPs associated with milk production traits.

**Trait**	**SNP name**	**SNP rs**	**Chromosome**	**Position**	**(Ref/Alt)**	**Effect**	**Nearest genes**	**Sig**
TP	BTA-87678-no-rs	rs41661899	6	9,493,248	G/A	−7.564	TRAM1L1	9.34e − 07

CN	BTB-01114242	rs42274954	12	4,125,837	G/A	1.704	DIAPH3	9.75e − 07

W	BTA-87678-no-rs	rs41661899	6	9,493,248	G/A	−2.22	TRAM1L1,	3.16e − 07

P	BovineHD4100006892	rs43560693	8	70,560,867	G/A	0.073	PEBP4	8.14e − 07
	ARS-BFGL-NGS-64829	rs109098713	10	76,196,982	G/A	0.07	WDR89	7.88e − 07
	ARS-BFGL-NGS-13077	rs111029661	14	12,413,503	A/G	0.184		5.06e − 13
	BovineHD1500003496	rs134499665	15	13,957,841	T/C	−0.074		7.49e − 08
	BovineHD1900003355	rs133908307	19	12,335,198	G/A	−0.163	BCAS3	4.61e − 07
	BovineHD2100013425	rs133627532	21	46,418,474	A/G	−0.128	RALGAPA1	6.18e − 07
	ARS-BFGL-NGS-3209	rs42098411	26	34,311,810	G/A	0.241	HABP2	3.34e − 10
	ARS-BFGL-NGS-36993	rs110066280	28	38,157,735	A/G	−0.114	NRG3	2.98e − 08

D	ARS-BFGL-NGS-32544	rs110844447	6	99,861,651	A/G	1.034	HPSE	2.90e − 07

S	BovineHD0600014178	rs109564259	6	51,439,843	G/G	−0.022	PCDH7	1.09e − 07
	BovineHD1100017863	rs135552551	11	63,021,847	A/G	−0.02	LINC02579	4.51e − 07
	Hapmap50890-BTA-121436	rs41620904	24	28,267,327	A/G	0.019	TRNAS-GGA	9.72e − 08

FP	BovineHD0600000010	rs135995768	14	17,128	A/G	−0.028	OR5CN1P	4.55e − 07

Abbreviations: CN, casein; D, density; FP, freezing point; P, protein percentage; S, salt; TP, total protein; W, whey.

## Data Availability

The dataset used in this study is available upon request from the corresponding author.

## Supporting Information

Supporting Information Additional supporting information can be found online in the Supporting Information section. Figure S1. Frequency distribution of transformed phenotypes for milk production traits in crossbred genotyped dairy cows. The figure displays the distributions for the following traits: (a) total protein content (TP), (b) casein content (CN), (c) whey protein (W), (d) protein percentage, (e) total daily milk yield (TDMY), (f) fat percentage (F), (g) lactose content (L), (h) total solids (TSs), (i) density (D), (j) solids-not-fat (SNF), (k) salt (S), and (l) freezing point (FP). These distributions demonstrate the variability and normality of each trait after transformation, facilitating the genomic analysis of milk production traits. The frequency histograms provide a visual representation of the underlying phenotypic data used for further genetic association studies. Table S1. SNP distribution across the 29 chromosomes in 308 crossbred genotyped dairy cows. The table presents the length of each chromosome in megabases (Mb), the number of single nucleotide polymorphisms (SNPs) identified, and the SNP density (number of SNPs per Mb). This information is critical for understanding the genomic coverage and marker density used in the genomic analyses. The SNP density is relatively uniform across chromosomes, ensuring that the genome-wide association study (GWAS) results are robust and comprehensive.
