# Supplementary material for: Genome-Wide Association Study for Test-Day Milk Yield, Proteins, and Composition Traits of Crossbred Dairy Cattle in Ethiopia
Source: Int J Genomics. 2024 Oct 21;2024:1472779. doi: 10.1155/2024/1472779 (PMC11519051; doi:10.1155/2024/1472779)
Supplement: Supporting Information — Additional supporting information can be found online in the Supporting Information section. Figure S1. Frequency distribution of transformed phenotypes for milk production traits in crossbred genotyped dairy cows. The figure displays the distributions for the following traits: (a) total protein content (TP), (b) casein content (CN), (c) whey protein (W), (d) protein percentage, (e) total daily milk yield (TDMY), (f) fat percentage (F), (g) lactose content (L), (h) total solids (TSs), (i) density (D), (j) solids-not-fat (SNF), (k) salt (S), and (l) freezing point (FP). These distributions demonstrate the variability and normality of each trait after transformation, facilitating the genomic analysis of milk production traits. The frequency histograms provide a visual representation of the underlying phenotypic data used for further genetic association studies. Table S1. SNP distribution across the 29 chromosomes in 308 crossbred genotyped dairy cows. The table presents the length of each chromosome in megabases (Mb), the number of single nucleotide polymorphisms (SNPs) identified, and the SNP density (number of SNPs per Mb). This information is critical for understanding the genomic coverage and marker density used in the genomic analyses. The SNP density is relatively uniform across chromosomes, ensuring that the genome-wide association study (GWAS) results are robust and comprehensive. [file 1472779.f1.docx]

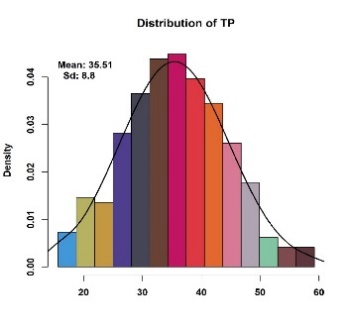


**(a)**


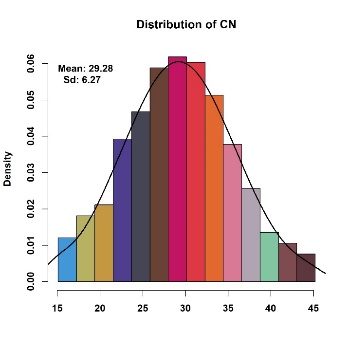


**(b)**


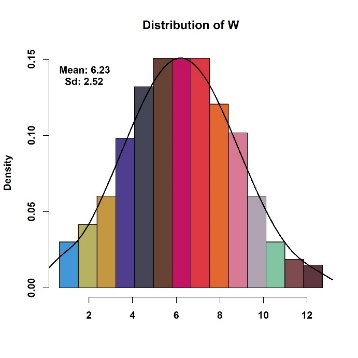


**(c)**


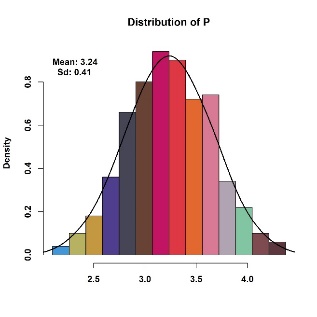


**(d)**


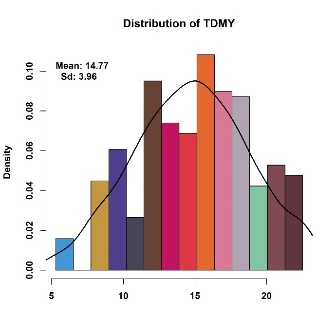


**(e)**


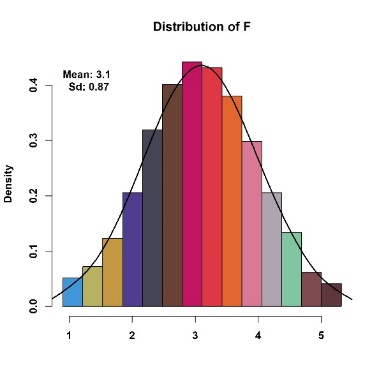


**(f)**


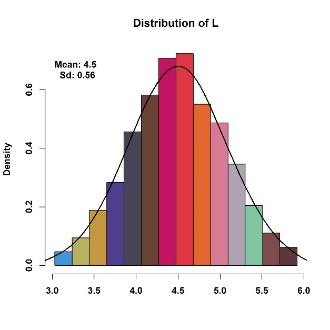


**(g)**


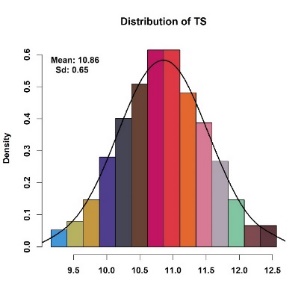


**(h)**


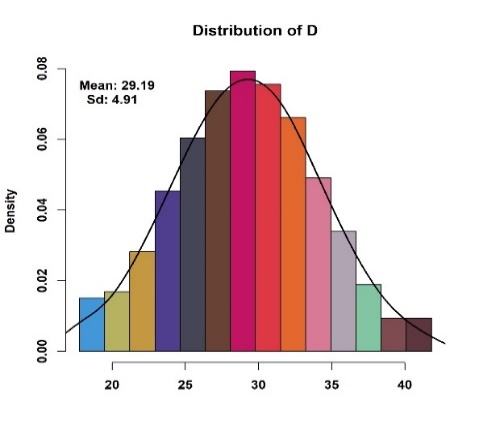


**(i)**


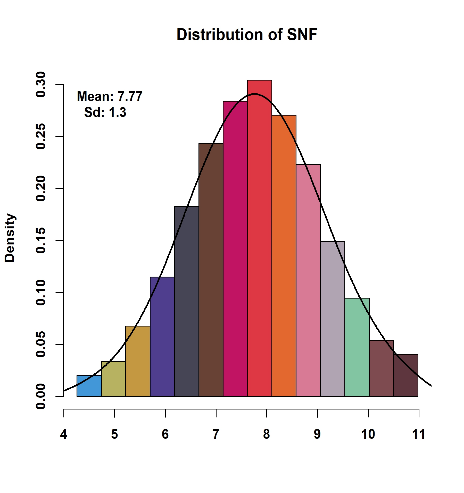


**(j)**


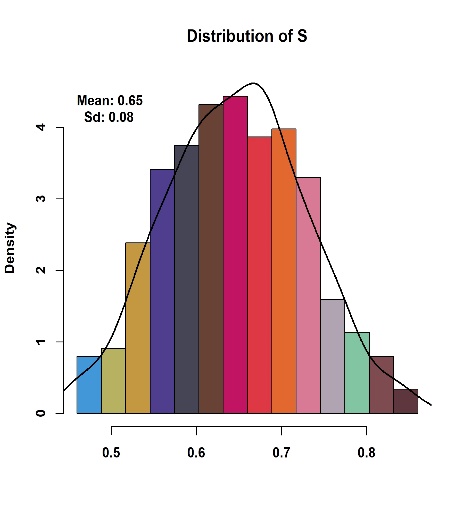


**(k)**


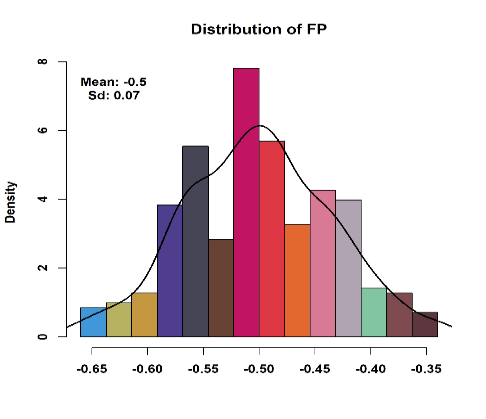


**(l)**

**Supplementary Figure 1.** Frequency distribution of transformed phenotypes for milk production traits in crossbred genotyped dairy cows. The figure displays the distributions for the following traits: (a) total protein content (TP), (b) casein content (CN), (c) whey protein (W), (d) protein percentage, (e) total daily milk yield (TDMY), (f) fat percentage (F), (g) lactose content (L), (h) total solids (TS), (i) density(D), (j) solids-not-fat (SNF), (k) salt (S), and (l) freezing point (FP). These distributions demonstrate the variability and normality of each trait after transformation, facilitating the genomic analysis of milk production traits. The frequency histograms provide a visual representation of the underlying phenotypic data used for further genetic association studies.

**Supplementary Table 1.** SNP distribution across the 29 chromosomes in 308 crossbred genotyped dairy cows**.** The table presents the length of each chromosome in megabases (Mb), the number of single nucleotide polymorphisms (SNPs) identified, and the SNP density (number of SNPs per Mb). This information is critical for understanding the genomic coverage and marker density used in the genomic analyses. The SNP density is relatively uniform across chromosomes, ensuring that the genome-wide association study (GWAS) results are robust and comprehensive.

| **Chromosome** | **Length (Mb)** | **SNPs** | **Density (SNPs/Mb)** |
| --- | --- | --- | --- |
| 1 | 158 | 5246 | 33.2 |
| 2 | 136 | 4476 | 32.91 |
| 3 | 123 | 4254 | 34.59 |
| 4 | 120 | 3830 | 31.92 |
| 5 | 121 | 4196 | 34.68 |
| 6 | 122 | 4098 | 33.59 |
| 7 | 112 | 3650 | 32.59 |
| 8 | 113 | 3631 | 32.13 |
| 9 | 105 | 3518 | 33.5 |
| 10 | 104 | 3467 | 33.34 |
| 11 | 107 | 3590 | 33.55 |
| 12 | 91 | 2866 | 31.49 |
| 13 | 84 | 2914 | 34.69 |
| 14 | 84 | 2898 | 34.5 |
| 15 | 85 | 2878 | 33.86 |
| 16 | 81 | 2670 | 32.96 |
| 17 | 75 | 2519 | 33.59 |
| 18 | 65 | 2419 | 37.22 |
| 19 | 63 | 2447 | 38.84 |
| 20 | 71 | 2604 | 36.68 |
| 21 | 71 | 2436 | 34.31 |
| 22 | 61 | 2093 | 34.31 |
| 23 | 52 | 1977 | 38.02 |
| 24 | 62 | 2144 | 34.58 |
| 25 | 42 | 1606 | 38.24 |
| 26 | 51 | 1714 | 33.61 |
| 27 | 45 | 1606 | 35.69 |
| 28 | 46 | 1645 | 35.76 |
| 29 | 51 | 1734 | 34 |
